# An Aid to Economy: The Importance of Analysing Expenditure

**Published:** 1913-09-27

**Authors:** 


					September 27, 1913. THE HOSPITAL 757
AN AID TO ECONOMY.
The Importance of Analysing Expenditure.
The analysis which is required by the Uniform
ystem of Accounts provides a ready and obvious
^eans of comparing expenditure between one
Pei iod and another in respect of the various head-
j^gs dealt with. It is not generally recognised,
I ?Wever, that this analysis may with advantage
carried a great deal further and the number of
fadings largely increased. No work in this
potion is wasted, for the more completely all
^ e details of expenditure are known the less likeli-
p??d is there of extravagance going unperceived.
.^travagance is not always wilful, for an increase
^ the consumption of stores may be so gradual as
escape notice, unless the quantities issued are
Quced to paper and the totals between one period
u another compared. There is scarcely a single
head on the debit side of the Income and Ex-
Qditure Account which does not permit of further
vision and sub-division. With regard to " Pro-
fl0ns," for instance, whether considered as a
?le or as separate items, there is an obvious
^alysis not provided for under the Uniform
?namely, that between staff and patients,
^nere the hospital is at all a large one, the
infmer ^ese divisions may be further divided
0 such headings as Medical Officers, Nurses,
0rHestics, and so on. Similarly, under " Surgery
nd Uispensaiy," the three items?Drugs, Chemi-
cals, Disinfectants?which are usually classed
together, may with advantage be kept distinct,
so that if an increase be noticed under the sub-
head as a whole, the weak spot may be at once
detected.
Tbacing Responsibility.
Where it is desired to trace the respon-
sibility to an individual, further divisions may be
made, such as Dispensary, Clinical Laboratory,
X-Ray Department, Anaesthetics, etc., etc. On
the surface it may appear that this involves a
great deal of work, but actually it is not so, for the
officer responsible for the invoices is usually in a
position to say exactly for whom the various articles
are intended; and, if he makes a rough note accord-
ingly, the way is clear for the invoices to be gone
through periodically, and the various forms entered
up. With regard to Instruments and Appliances, a
valuable comparison will be provided if the repairs
and renewals are analysed under the different
wards and theatres. Under the heading of
" Domestic," sub-divisions of the sub-heads will
readily suggest themselves, principally in con-
nection with the items?Coal, Gas, Electric
Current, and Uniforms. The remaining headings
of the Income and Expenditure Account will bear
analysis proportionately to the size and circum-
stances of the hospital concerned.
Coal.
Comparison of Consumption and Cost in 1912 and 1913.
First Half-year.
Hilary 1912 ...
.. 1913 ...
Urease or Decrease
>4t*Uary 1912 ...
1913 ...
Urease or
*Wh 1912 ...
? 1913
Urease or Decrease
W. or Dec. for Quarter
^PHl 1912
?< 1913
^crease or Decrease ...
1912
? 1913
^crease or Decrease ...
1912
?? 1913
Urease or Decrease ...
lnc. or Dec. for Quarter
or Dec. for Half-year
Tons.
House.
+ 12
+ 30
- 22
+ 20
- 12
- 18
+ 2
Steam.
113
- 25
145
134
- 11
- 20
Cost.
House.
? S. d.
15 4 6
26 0 0
+ 10 15 6
30 9 0
57 4 0
+ 23 15 0
60 18 0
40 10 0
- 20 8 0
4- 17 2 6
23 6 0
12 12 0
- 10 14 0
10 3 0
5 4 0
- 4 19 0
5 1 6
5 4 0
+- 2 6
- 12 10 6
I + 1 12 0
I
Steam.
? s. d.
85 4 5
79 18 8
- 5 5 9
116 4 5
121 14 4
+ 5 9 11
74 9 8
70 1 6
-482
-440
74 9 8
65 2 8
-970
54 10 0
47 4 8
-754
31 19 3
29 1 3
- 2 18 0
-19 10 4
-23 14 4
Second Half-year.
July 1912
1913
Increase or Decrease ...
August 1912
? 1913
Increase or Decrease ...
September 1912
1913
Increase or Decrease ...
Inc. or Dec. for Quarter
October 1912
? 1913
Increase or Decrease ...
November 1912
1913
Increase or Decrease ...
December 1912 ...
1913
Increase or Decrease ...
Inc. or Dec. for Quarter
Inc. or Dec. for Half-year
Tons.
House.
26
28
34
Steam.
42
26
30
46
72
108
Cost.
House.
? s. d.
5 4 0
22 12 4
24 5 4
29 9 4
Steam.
a s. d.
36 10 6
23 12 4
27 5 0
41 15 8
65 6 4
3 8
Suggested Form for Checking Coal Consumption.
758 THE HOSPITAL September 27, 1913-
For the proper control of expenditure, it is not
sufficient that the extended analyses here referred
io should be done spasmodically, or merely when
an increase has become apparent in some direction
or other. The records and comparisons should be
systematic and continuous, so that they may be
??taken up and found of value at almost any moment.
To enable this to be achieved, it is necessary that
the forms requisite for the purpose should be
drawn up with care and foresight. They should
be arranged to last for twelve months, so that they
may all be prepared once a year only. Each item
of expenditure requires to be dealt with separately,
and when the matter is tackled for the first time
much thought and ingenuity will be found neces-
sary before a form is drawn up that embodies all
the information required, and is yet compact and
?concise. As an example, the accompanying form in
connection with the comparatively simple matter
of Coal may be given. Such a form, as may "e.
seen, shows not only the quantities of House a_n
Steam Coal consumed, but their values, and it lD'
dicates at a glance the increase or decrease Pe!j
month, per quarter, and per half-year. In actua
practice, the increases would be shown in red 111
and the decreases in black ink.
When all the forms have been drawn up,
details may be filled in from time to time, aS
already stated, direct from the invoices. The
forms may then be submitted to the Secretary
to the House Committee, who are thereby furnishe
with a ready and invaluable means of controlllD?
the expenditure. No doubt the preparation an
upkeep of the records will entail a certain amouD
of extra work on the clerical staff, but when onc?
the matter has been placed upon a systematic basis>
it will soon come to be regarded as part of the usua
routine.

				

